# Effect of pyloroplasty on clinical outcomes following esophagectomy

**DOI:** 10.1007/s00464-024-11265-0

**Published:** 2024-10-04

**Authors:** Sophie L. F. Doran, Maria G. Digby, Sophie V. Green, Clive J. Kelty, Anand P. Tamhankar

**Affiliations:** 1 Department of Upper Gastrointestinal Surgery, Sheffield Teaching Hospitals NHS Foundation Trust, Sheffield, S5 7AU UK; 2 Academic Unit of Surgery, University of Sheffield, Northern General Hospital, Herries Road, Sheffield, S5 7AU UK

**Keywords:** Esophagectomy, Pyloroplasty, Weight loss

## Abstract

**Introduction:**

The role of concurrent pyloroplasty with esophagectomy is unclear. Available literature on the impact of pyloroplasty during esophagectomy on complications and weight loss is varied. Data on the need for further pyloric intervention are scarce. Our study compares the clinical outcomes after esophagectomy with or without pyloroplasty and investigates the role of post-operative pyloric dilatation.

**Methods:**

Consecutive patients (*n* = 207) undergoing Ivor Lewis esophagectomy performed by two surgeons at our institution were included. Data on patient demographics, mortality rate, anastomotic leak, respiratory complications (Clavien-Dindo grade ≥ 3), anastomotic stricture rate, and percentage weight loss at 1 and 2 year post-operatively were evaluated. For weight analysis at 1 and 2 year post-operatively, patients were excluded if they had been diagnosed with recurrence or died prior to the 1 or 2 year timepoints.

**Results:**

Ninety-two patients did not have a pyloroplasty, and 115 patients had a pyloroplasty. There were no complications resulting from pyloroplasty. There was no significant demographic difference between the groups except for age. Mortality rate, anastomotic leak, respiratory complications, anastomotic stricture rate, and percentage weight loss at 1 and 2 years were statistically similar between the two groups. However, 14.1% of patients without pyloroplasty required post-operative endoscopic pyloric balloon dilatation to treat respiratory complications or gastroparesis. Subgroup analysis of patients without pyloroplasty indicated that patients requiring dilatation had greater weight loss at 1 year (15.8% vs 9.4%, *p* = 0.02) and higher respiratory complications rate (27.3% vs 4.7%, *p* = 0.038).

**Conclusions:**

Overall results from our study that pyloroplasty during Ivor Lewis esophagectomy is safe and useful to prevent the need for post-operative pyloric dilatation.

The Ivor Lewis esophagectomy is the most commonly employed surgical approach for the treatment of potentially curable esophageal and gastro-esophageal junction carcinoma. There has been a steady improvement in survival following radical treatment for esophageal carcinoma with Ivor Lewis esophagectomy due to earlier detection, optimization of surgical technique and peri-operative management, and improvement in peri-operative chemo/radiotherapy regimes [1, 2]. This has led to a significant increase in both overall and disease-free survival, with 40% of patients alive up to 5 years following radical surgery [3]. Despite these improvements, Ivor Lewis esophagectomy remains a complex multi-step procedure with complication rates which are higher than most other surgical procedures [4]. This has resulted in an increasing emphasis now on the importance of improving post-operative outcomes and quality of life by reducing post-operative complications following esophagectomy.

The optimal management of the pylorus during esophagectomy and its impact on post-operative outcomes remains a matter for debate in the surgical community. Historically the impetus to perform a pyloric drainage procedure during esophagectomy is originated from experience with truncal vagotomy for peptic ulcer disease [5]. Proponents of pyloric intervention advocate that the high thoracic bilateral vagotomy required for oncological quality leads to pyloric denervation, gastric dysmotility, and ultimately delayed gastric emptying with the associated sequelae of aspiration pneumonia, nausea, vomiting, and increased risk of anastomotic leakage [6, 7]. Opponents of pyloric intervention advocate that delayed gastric emptying does not impact on all patients, and most patients respond to medical therapy and (or) endoscopic pyloric dilatation [8–10]. Studies also suggest that delayed gastric emptying may recover with time following surgery as new migrating motor complexes are produced within the gastric remnant which in turn restores gastric contractility and pyloric function. Further arguments against pyloric intervention also include the risk of the procedure itself including leakage, increase in operative time (particularly in the era of minimally invasive esophagectomy) and long-term sequelae such as bile reflux.

The true prevalence of delayed gastric emptying following esophagectomy is unknown—there is a wide variation in incidence from 4 to 62% reported in the literature [11–15]. This is likely related to a lack of a standard definition for delayed gastric emptying which further complicates comparison and interpretation of results from different studies [16]. Delayed gastric emptying after esophagectomy essentially implies a stagnant immotile gastric conduit. This has the potential of causing conduit distention, anastomotic distraction and leak, aspiration and respiratory complications, reflux and anastomotic strictures as well as long-term weight loss. These complications are often used as surrogate markers of delayed gastric emptying and post-operative pyloric function. However, available literature on the impact of pyloroplasty during esophagectomy on complications and weight loss is varied and inconsistent.

Data on outcomes in patients who do not have pyloric drainage during esophagectomy but require post-operative pyloric intervention are scarce. Optimum management of pylorus during or after esophagectomy has the potential of improving post-esophagectomy outcomes but continues to remain a divisive issue in the surgical community.

The aim of this study is to compare the clinical outcomes including weight loss after esophagectomy with or without pyloric intervention in the form of pyloroplasty and investigate the role of post-operative pyloric dilatation.

## Methods

### Study design

This was a retrospective analysis of a contemporaneously maintained database at a regional upper gastro-intestinal cancer center in the United Kingdom. All patients who underwent an Ivor Lewis esophagectomy for adenocarcinoma or squamous cell carcinoma of the esophagus or gastro-esophageal junction between April 2013 and April 2021 were included. Data collection included patient demographics (age, gender, ASA (American Society of Anesthesiologists) grade, neoadjuvant treatment, and pre-operative weight). Post-operative histological parameters were collected including tumor subtype (adenocarcinoma or squamous cell carcinoma), tumor site, tumor, lymph node, metastasis, and resection margin stage according to the 8th edition of the American Joint Committee on Cancer (AJCC) staging of epithelial cancers of the esophagus and esophagogastric junction. Patients were divided into two groups by pyloric intervention, including no pyloroplasty and those that had a pyloroplasty.

Clinical outcomes including 30 and 90 days mortality rate, anastomotic leak rate, chyle leak rate, respiratory complication rate, esophageal anastomotic stricture rate, and percentage weight loss at 1 year and 2 years were collected, and rate of post-operative pyloric intervention in the form of endoscopic pyloric balloon dilatation was collected.

### Surgical technique

Each patient underwent clinical staging using a combination of endoscopy, computed tomogram, and positron emission tomogram as is the standard at our institution. Neoadjuvant therapy with chemotherapy or chemoradiotherapy was given to all patients with node-positive disease and/or ≥ T2 disease. Resection was performed 5–8 weeks following completion of neoadjuvant therapy. All patients included in the study were referred to our unit for esophagectomy and assigned to one of the two surgeons. Patients were assigned on a consecutive rotational basis without any selection bias. All operations were performed by one of the two surgeons (denoted surgeon A and surgeon B) within the Department of Upper Gastrointestinal Surgery.

A standard Ivor Lewis esophagectomy was performed using trans-abdominal and right thoracotomy access. A complete lymphadenectomy of the celiac branches was performed. The stomach and distal esophagus were mobilized to above the hiatus. At the end of the abdominal phase, surgeon A did not routinely perform a pyloroplasty and surgeon B routinely did perform a pyloroplasty. During pyloroplasty, the entire muscle and gastric mucosa at the pylorus were divided longitudinally. The pylorotomy was then closed transversely in a single layer with interrupted 3–0 polydioxanone sutures to create a Heineke–Mikulicz type pyloroplasty. Feeding jejunostomies were not routinely inserted. Thoracic esophageal mobilization and lymphadenectomy were then performed via a right posterolateral approach. Azygos arch was divided and thoracic duct ligated above the diaphragm. The stomach was tubularized to create a conduit approximately 5 cm in width, and a subsequent intrathoracic anastomosis using a circular stapler was performed above the level of azygos arch. All patients were managed according to a standardized post-operative protocol. All patients received proton pump inhibitor therapy post-operatively and was continued on a long-term basis orally after discharge.

### Definitions

Mortality rate: patients who did not survive 30 day or 90 days time points were identified for mortality rate assessment.

Anastomotic leak: Anastomotic leak was diagnosed by oral contrast study and computed tomogram following clinical suspicion as indicated by fever and/or leucocytosis or rising C reactive protein level.

Respiratory complications: Respiratory complications, including pneumonia (diagnosed by a combination of clinical symptoms suggestive of the diagnosis, leukocytes, and infiltrates on imaging), were recorded if classified as Clavien–Dindo grade ≥ 3.

Anastomotic stricture: Anastomotic stricture was identified in the presence of dysphagia and stenosis at the anastomosis on oral contrast studies or endoscopy and required endoscopic dilatation.

Post-operative pyloric intervention: Patients were offered endoscopic dilatation of the pylorus when they had evidence of delayed transit of oral contrast on imaging studies and had symptoms of conduit dysfunction or delayed gastric emptying (inadequate oral intake, early satiety, nausea, and vomiting) causing nutritional impairment or in cases with severe respiratory complications with a dilated non-draining conduit. Patients with severe respiratory complications without a dilated conduit were not offered pyloric dilatation.

Percentage weight loss: Weight loss at 1 and 2 year time points was compared to immediate pre-operative weight (following any neoadjuvant treatment) to calculate percentage weight loss.

### Exclusion criteria

Patients were excluded from analysis if they underwent an Ivor Lewis esophagectomy for a diagnosis other than adenocarcinoma or squamous cell carcinoma of the esophagus or gastro-esophageal junction. For the evaluation of percentage weight loss at 1 and 2 years post-operatively, patients were excluded if they had been diagnosed with recurrence or died prior to the 1 or 2 year timepoints.

### Statistical analysis

Demographic data were summarized and compared between the pyloric intervention groups using the Mann–Whitney *U-*test for continuous variables and Fisher’s exact test or Chi Squared test as appropriate for categorical variables. For all tests, a two-sided p value of 0.05 was deemed to be significant. All statistical analysis was conducted on DATAtab: Online Statistics Calculator (DATAtab e.U. Graz, Austria).

### Ethics and consent

Data analyzed in this study were used from a contemporaneously maintained database from the Upper Gastrointestinal Surgical unit at our institute. Data review was approved by Local Clinical Effectiveness Unit (CEU project registration number: 11763). No patient identifying information was recorded, and patient consent was not required for data review as per CEU guidelines.

## Results

During the study period, 207 patients met the inclusion criteria. 92 patients (44.4%) did not have a pyloroplasty (group A), and 115 patients (55.6%) had a pyloroplasty (group B). There were no complications resulting from pyloroplasty. There was no statistically significant difference between the groups in terms of gender, ASA grade, pre-operative weight, neoadjuvant treatment, tumor subtype, tumor site, TNM stage, or resection margin status. Of note, all patients were classified as ASA grade 2 or 3, and all patients were M0 according to the TNM system. There were no R1 longitudinal or R2 resections. The median age was younger in the pyloroplasty group. All results are summarized in Table 1.

**Table 1:** Patient Characteristics

	Group A	Group B	*p* value^*a*^
	No pyloroplasty	Pyloroplasty	
	(*n* = 92)	(*n* = 115)	
Age	69 (62–72)	65 (59–71)	0.045^b,^*
Gender			0.258
Male	77 (83.7%)	89 (77.4%)	
Female	15 (16.3%)	26 (22.6%)	
ASA grade			0.469
2	72 (78.3%)	85 (73.9%)	
3	20 (21.7%)	30 (26.1%)	
Pre-operative weight (kg)	77.0 (69.9–87.1)	80.2 (69.8–91.0)	0.244^b^
Neoadjuvant treatment			0.947
Yes	62 (67.4%)	77 (67.0%)	
No	30 (32.6%)	38 (33.0%)	
Tumor subtype			0.484
Squamous cell carcinoma	7 (7.6%)	12 (10.4%)	
Adenocarcinoma	85 (92.4%)	103 (89.6%)	
Tumor site			0.782
Mid esophagus	6 (6.5%)	6 (5.2%)	
Lower esophagus	54 (58.7%)	64 (55.7%)	
Gastroesophageal junction	32 (34.8%)	45 (39.1%)	
T stage			0.636
0–1	25 (27.2%)	40 (34.8%)	
2	15 (16.3%)	14 (12.2%)	
3	50 (54.3%)	59 (51.3%)	
4	2 (2.2%)	2 (1.7%)	
N stage			0.149
0	38 (41.3%)	60 (52.2%)	
1	28 (30.4%)	26 (22.6%)	
2	19 (20.7%)	15 (13.0%)	
3	7 (7.6%)	14 (12.2%)	
Resection margin			0.170
R0	67 (72.8%)	93 (80.9%)	
R1 (circumferential)	25 (27.2%)	22 (19.1%)	

Values are median (IQR), otherwise n. ^a^Chi-Squared test except ^b^Mann-Whitney U-Test

*Denotes statistical significance at 0.05 level

### Analysis of clinical outcomes

Thirty day and 90 days mortality rate, anastomotic leak rate, chyle leak rate, respiratory complications (Clavien-Dindo grade ≥ 3), and anastomotic stricture rates were similar in both groups. Percentage weight loss at 1 and at 2 years was also similar. These results are summarized in Table 2.

**Table 2:** Clinical outcomes

	Group A no pyloroplasty (*n* = 92)	Group B pyloroplasty (*n* = 115)	*p* value^a^
30 day mortality	1 (1.1%)	3 (2.6%)	0.429
90 day mortality	4 (4.3%)	4 (3.5%)	0.747
Anastomotic leak	5 (5.4%)	15 (13.0%)	0.066
Chyle leak	5 (5.4%)	3 (2.6%)	0.295
Respiratory complications	7 (8.2%)	12 (11.7%)	0.484
Anastomotic stricture	20 (21.7%)	16 (13.9%)	0.140
Weight loss at 1 year	10.2% (3.1–16.2, *n* = 74)	12.6% (8.0–17.3, *n* = 88)	0.072^b^
Weight loss at 2 years	8.8% (0.3–16.2, *n* = 49)	10.5% (6.8–17.5, *n* = 68)	0.159^b^
Required pyloric dilatation	13 (14.1%)	0 (0.0%)	< 0.001*

Values are median (IQR), otherwise n. ^a^Chi-Squared test except ^b^Mann-Whitney U-Test

*Denotes statistical significance at 0.05 level

### Subgroup analysis of patients not having pyloroplasty

A subgroup analysis was performed of patients who required post-operative pyloric intervention. There were no patients in the pyloroplasty group who required pyloric intervention post-operatively, but 14.1% of patients without pyloroplasty required post-operative endoscopic pyloric balloon dilatation (*p* < 0.001). 21 dilatations in 13 patients in the no pyloroplasty group were performed to treat symptoms or complications caused due to delayed gastric emptying.

Subgroup analysis of group A (no pyloroplasty group) was performed. There was no statistically significantly difference between those that did not require a pyloric balloon dilatation and those that did in terms of age, gender, ASA grade, pre-operative weight, neoadjuvant treatment, tumor subtype, tumor site, T stage, or resection margin status. The group that required a pyloric balloon dilatation was more likely to have N stage ≥ 1. These results are summarized in Table 3.

**Table 3:** Patient Characteristics of group A (no pyloroplasty group)

	No dilatation (*n* = 79)	Dilatation (*n* = 13)	*p* value^a^
Age	68 (60–72)	70 (65–73)	0.304^b^
Gender			0.476
Male	67 (84.8%)	10 (76.9%)	
Female	12 (15.2%)	3 (23.1%)	
ASA grade			0.115
2	64 (81.0%)	8 (61.5%)	
3	15 (19.0%)	5 (38.5%)	
Pre-operative weight (kg)	76.0 (68.5–89.0)	80.0 (75.8–85.4)	0.564^b^
Neoadjuvant treatment			0.261
Yes	55 (69.6%)	7 (53.8%)	
No	24 (30.4%)	6 (46.2%)	
Tumor subtype			0.254
Squamous cell carcinoma	5 (6.3%)	1 (7.7%)	
Adenocarcinoma	74 (93.7%)	12 (92.3%)	
Tumor site			0.927
Mid esophagus	5 (6.3%)	1 (7.7%)	
Lower esophagus	47 (59.5%)	7 (53.8%)	
Gastroesophageal junction	27 (34.2%)	5 (38.5%)	
T stage			0.654
0–1	24 (30.4%)	2 (15.4%)	
2	12 (15.2%)	3 (23.1%)	
3	42 (53.2%)	8 (61.5%)	
4	1 (1.2%)	0 (0.0%)	
N stage (all stages)			0.003*
0	36 (45.6%)	2 (15.4%)	
1	23 (29.1%)	5 (38.4%)	
2	17 (21.5%)	2 (15.4%)	
3	3 (3.8%)	4 (30.8%)	
N stage (N0 vs N ≥ 1)			
0	36 (45.6%)	2 (15.4%)	0.041*
≥ 1	43 (54.4%)	11 (84.6%)	
Resection margin			0.324
R0	59 (74.7%)	8 (61.5%)	
R1 (circumferential)	20 (25.3%)	5 (38.5%)	

Values are median (IQR), otherwise n.^a^Chi-Squared test except ^b^Mann- Whitney U-Test

*Denotes statistical significance at 0.05 level

A further subgroup analysis of Group A (no pyloroplasty) indicated that patients requiring endoscopic pyloric balloon dilatation had a higher respiratory complication rate (Clavien-Dindo grade ≥ 3) and greater weight loss at 1 year. However, patients who required a pyloric balloon dilatation did not have a higher 30 or 90 days mortality rate, anastomotic leak, chyle leak or stricture rate, and by 2 years post-operatively, there was no statistically significant difference in percentage weight loss. These results are summarized in Table 4.

**Table 4:** Subgroup analysis of group A (no pyloroplasty group)

	No dilatation (*n* = 79)	Dilatation (*n* = 13)	*p* value
30 day mortality	1 (1.3%)	0 (0.0%)	0.683^c^
90 day mortality	3 (3.8%)	1 (7.7%)	0.523^c^
Anastomotic leak	5 (6.3%)	0 (0.0%)	0.351^c^
Chyle leak	5 (6.3%)	0 (0.0%)	0.351^c^
Respiratory complications	4 (5.1%)	3 (23.1%)	0.023^c,^*
Anastomotic stricture	18 (22.8%)	2 (15.4%)	0.549^c^
Weight loss at 1 year	9.4% (2.8–15.5, *n* = 63)	15.8% (9.7–21.6, *n* = 11)	0.020^b,^*
Weight loss at 2 years	8.3% (0.1–16, *n* = 44)	13.4% (8.2–22.4, *n* = 5)	0.159^b^

^b^Mann-Whitney U-test ^c^Fisher’s exact test

*Denotes statistical significance at 0.05 level

## Discussion

The role of concurrent pyloroplasty with esophagectomy has been long debated. Vagal parasympathetic supply causes peristalsis in the stomach and relaxation of the pylorus. So, performing a pyloroplasty with esophagectomy where both vagi are divided is intuitive to improve gastric drainage, which in-turn could reduce gastric conduit distension, anastomotic distraction, aspiration, and improve nutrition. Early research from the 1980s did show that there was reduction in delayed gastric emptying and complications after esophagectomy when pyloric drainage was performed [17, 18]. Harada and colleagues in their study also showed that patients with concurrent pyloroplasty lose less weight after esophagectomy at one year [19].

However, pyloric drainage is not universal after esophagectomy. A large proportion of surgeons do not believe that the absence of pyloroplasty causes enough delayed gastric emptying to result in excess complications following an esophagectomy. Doing a pyloroplasty could also add to operative time, bile reflux, and complications from pyloroplasty. Several meta-analyses have demonstrated that there was no difference in the rates of respiratory complications, anastomotic leak and mortality between treatment and no treatment of the pylorus during esophagectomy [15, 16, 20]. More recent meta-analyses by Nevins et al. [21] analyzing 2339 patient and by Loo et al. [22] analyzing 1164 patients have concluded that adding a pyloric drainage procedure did not reduce post-operative complications after esophagectomy.

It is, however, well accepted that all of the studies used in these meta-analyses suffer from heterogeneity in both the definition and assessment of delayed gastric emptying, the definition of complications such as anastomotic leak and respiratory complications and most importantly the technique of pyloric intervention used. These studies often combine various different techniques of pyloric drainage including pyloromyotomy, pyloroplasty, intra-operative balloon dilatation, botulinum toxin injection, or even digital dilatation. We believe that pyloroplasty is the gold standard pyloric drainage procedure as compared to pyloromyotomy, pyloric balloon dilatation, or botulinum toxin injections which all may have a variable effect on completely destroying the pyloric sphincter.

In addition, the numbers of specific pyloric drainage interventions in individual papers assessed in these meta-analyses are often very small. None of the individual papers included in the meta-analyses have over 100 pyloroplasties in the intervention arm.

Our study is a large single-center series looking at the effect of pyloroplasty or not doing it on post-operative complications, and outcomes include weight loss. All investigated patients in the study were operated on by one of two surgeons using the same resection and reconstruction principle in addition to a standardized post-operative protocol. Intraoperative pyloric drainage was consistently a standard pyloroplasty and post-operative intervention when required was with an endoscopic balloon dilatation. Pyloroplasty is well established as a low-risk procedure, and we had no complications from pyloroplasty in our study. Although our study did not show any actual difference in mortality, anastomotic leak, severe respiratory complications, anastomotic strictures, or weight loss between patients with or without pyloroplasty, we have demonstrated that there exists a subset of patients (over 14%) who suffer from the absence of pyloric drainage (pyloroplasty). These patients required post-operative pyloric dilatations, had higher rate of respiratory complications and excessive weight loss at 1 year.

We analyzed the non-pyloroplasty subgroup and found that those needing pyloric dilatation had higher proportion of severe peri-operative respiratory complications and higher overall weight loss at 1 year. This is intuitive as if there was delayed gastric emptying it would naturally result in a higher aspiration risk and poorer nutrition. We were unable to do a multi-variable regression analysis for these two outcomes as the total number of patients with severe respiratory complications in the group was seven and weight loss was a continuous variable. It is possible to imagine that severe respiratory complications were more frequent in the dilatation group because one of the indications for a dilatation was severe respiratory complications. However, only 3 out of 7 patients who had severe respiratory complications in the no pyloroplasty group were offered dilatation as those without co-existent conduit distension were not offered intervention.

As a retrospective study, there are some limitations. In our study, we have used ASA grade as a surrogate for comorbidities. All patients were either ASA 2 or 3, and there was no statistically significant difference between the groups in terms of ASA grade. We also do not have exact recordings of operative time and blood loss. However, from our records, we have required peri-operative blood transfusion in less than two percent cases. As our study includes more than 200 patients, we do not feel that the mean or median of operative blood loss would differ between the two groups as the overall blood loss was generally very low. All patients included in the study were referred to our unit for esophagectomy and assigned to one of the two surgeons. Patients were assigned on a consecutive rotational basis without any selection bias. As our study group is over 200 patients and the patients were assigned to the surgeon on a rotational basis, we do not anticipate any selection bias with comorbidities.

Our results confirm that a subset of patients (over 14%) do fair badly in the absence of pyloroplasty. It has been demonstrated in previous studies that symptoms and sequelae of delayed gastric emptying following esophagectomy can be effectively and safely treated with endoscopic pyloric balloon dilatation [23, 24]. However, these patients are not treated until symptoms of delayed gastric emptying develop, and often secondary complications of delayed gastric emptying occur. This has been reinforced by the results of our study as despite judicious post-operative management and early endoscopic intervention for symptoms of delayed gastric emptying, there is an excess risk of secondary respiratory complications and excess weight loss at 1- year post-operatively.

Our overall major outcome results align well with similar results from various other studies and meta-analyses comparing pyloric drainage versus no drainage during an esophagectomy. We believe that Ivor Lewis esophagectomy is a complex, multi-step, multi-variable procedure and complications and outcomes do not necessarily depend on one single factor of pyloric drainage. Overall outcomes depend on the entire process including patient selection, prehabilitation, operative technique, and detail, as well as standardized enhanced recovery protocols. It would be too simplistic to accept that the single step of pyloroplasty would impact overall major outcomes. However, we have demonstrated that over 14% of patients who do not have pyloric drainage at the time of surgery in the form of a pyloroplasty will subsequently require an endoscopic pyloric balloon dilatation and still have a detrimental outcome. There were no adverse events related to intra-operative pyloroplasty. It may be beneficial to perform a pyloroplasty in all patients to avoid the requirement for post-operative endoscopic pyloric balloon dilatation and most importantly minimize the risk of respiratory complications and excess weight loss.

## Conclusion

Overall results from our study that pyloroplasty during Ivor Lewis esophagectomy is safe and useful to prevent the need for post-operative pyloric dilatation.
